# Mediterranean-Style Diet for the Primary and Secondary Prevention of Cardiovascular Disease: A Cochrane Review

**DOI:** 10.5334/gh.853

**Published:** 2020-08-12

**Authors:** Karen Rees, Andrea Takeda, Nicole Martin, Leila Ellis, Dilini Wijesekara, Abhinav Vepa, Archik Das, Louise Hartley, Saverio Stranges

**Affiliations:** 1 Division of Health Sciences, Warwick Medical School, University of Warwick, Coventry, UK; 2 Instiutute of Health Informatics Research, University College London, London, UK; 3 RTI Health Solutions, Manchester, UK; 4 Department of Epidemiology and Biostatistics, Schulich School of Medicine and Dentistry, Western University, London, CA; 5 Department of Family Medicine, Schulich School of Medicine and Dentistry, Western University, London, CA; 6 Department of Population Health, Luxembourg Institute of Health, Strassen, LU

**Keywords:** Mediterranean dietary pattern, Cardiovascular disease, Cardiovascular risk factors, Primary Prevention, Secondary Prevention, Systematic Review

## Abstract

**Background:**

Diet plays a major role in cardiovascular disease (CVD) risk.

**Objectives:**

To determine the effectiveness of a Mediterranean-style diet for the primary and secondary prevention of CVD.

**Methods:**

We searched for randomised controlled trials (RCTs) of Mediterranean-style diets in healthy adults and those at increased risk of CVD (primary prevention) and with established CVD (secondary prevention).

**Results:**

Thirty RCTs were included, 22 in primary prevention and eight in secondary prevention. Clinical endpoints were reported in two trials where there was moderate quality evidence for a reduction in strokes for primary prevention, and low quality evidence for a reduction in total and CVD mortality in secondary prevention. We found moderate quality evidence of improvement in CVD risk factors for primary prevention and low quality evidence of little or no effect in secondary prevention.

**Conclusions:**

There is still some uncertainty regarding the effects of a Mediterranean-style diet in CVD prevention.

## Introduction

Cardiovascular disease (CVD) is currently the leading cause of mortality worldwide, causing one-third of deaths globally [1]. Preventative strategies focus on modifiable risk factors such as high blood pressure, high cholesterol, smoking, obesity and poor diet, which account for a large proportion of the overall CVD burden [1], with poor diet estimated to be responsible for the largest contribution in Europe [2].

The Seven Countries study from the 1960s demonstrated the potential beneficial effects of a Mediterranean dietary pattern [3]. Systematic reviews of prospective observational studies have confirmed that greater adherence to a Mediterranean diet is associated with a significant improvement in health status and a significant reduction in overall mortality, as well as in morbidity and mortality from CVD and other major chronic diseases [4,5,6,7,8,9]. Benefits have also been found for individual CVD risk factors [10,11]. There is less evidence however from well-conducted and adequately powered randomised controlled trials (RCTs).

Studies also differ in their definition of a Mediterranean diet. The original description reflects the common dietary pattern of communities in countries of the Mediterranean region in the early 1960s [3], which was an expression of common cultural and historical roots, and a shared set of lifestyle and eating habits rather than a mere assortment of specific micro- and macro-nutrients [12]. The Mediterranean diet has been defined [13,14,15,16], and includes the following dietary factors: a high intake of plant foods comprising mainly fruits and vegetables, cereals and whole-grain breads, beans, nuts and seeds; locally grown, fresh and seasonal, unprocessed foods; large quantities of fresh fruit consumed daily whereas concentrated sugars or honey are consumed a few times per week in smaller quantities; olive oil as a main cooking ingredient and source of fat; low to moderate amounts of cheese and yogurt; low quantities of red meat and higher quantities of fish; and low to moderate amounts of red wine often accompanying main meals.

There is evidence supporting potential mechanisms to explain the beneficial effect of the Mediterranean diet on cardiovascular health [17]. Favourable effects have been shown on insulin resistance and endothelium-dependent vasoreactivity, as well as the antioxidant and anti-inflammatory effects of the Mediterranean diet and its individual components [18,19,20,21,22,23]. In addition, the Mediterranean dietary pattern has been associated with beneficial effects on many cardiovascular risk factors, including lipoproteins, obesity, diabetes mellitus and hypertension [10,11,24,25,26,27,28]. The protective effects of a Mediterranean-style diet on health outcomes are likely derived from synergistic interactions among different components as a whole dietary pattern rather than from relative effects of specific food groups [5]. The objectives of this review were to synthesise evidence from RCTs to determine the effectiveness of advice to follow a Mediterranean-style diet or provision of foods relevant to a Mediterranean-style diet, or both, on clinical endpoints and cardiovascular risk factors for the primary and secondary prevention of CVD [29].

## Methods

### Eligibility criteria

We included RCTs with follow-up periods of three months or more, examining the effects of a Mediterranean-style diet in adults (18 years or more) without established CVD and in those with established CVD to determine the effects of the intervention on primary and secondary prevention respectively. To reach our definition of a Mediterranean-style diet the intervention needed to include at least the following two components: a high monounsaturated/saturated fat ratio (use of olive oil as main cooking ingredient and/or consumption of other traditional foods high in monounsaturated fats such as tree nuts); and high intake of plant-based foods, including fruits, vegetables and legumes. This definition was based on recent work showing that the protective effects of the diet appear to be most attributable to olive oil, fruits, vegetables and legumes [5,30]. We considered trials where the comparison group was no intervention or minimal intervention (e.g. leaflet to follow a dietary pattern with no person-to-person intervention or reinforcement) and also other dietary interventions. Outcomes included CVD clinical endpoints and CVD risk factors (lipid levels, blood pressure).

### Information sources and search strategy

We identified trials through systematic searches of the following bibliographic databases (from inception to 26 September 2018): Cochrane Central Register of Controlled Trials, MEDLINE, EMBASE, and Web of Science Core Collection. We adapted the preliminary search strategy for MEDLINE (Ovid) for use in the other databases (Appendix 1). In addition, we checked reference lists of reviews for additional studies. We searched ClinicalTrials.gov and the World Health Organization International Clinical Trials Registry Platform (ICTRP) for ongoing trials. We applied no date or language restrictions. We contacted authors where necessary for further information.

### Study selection

Two review authors (of KR, NM, AT, LE, DW, AV, AD) independently screened titles and abstracts for inclusion of all the potential studies identified as a result of the searches and coded them as ‘retrieve’ (eligible or potentially eligible/unclear) or ‘do not retrieve’. We combined the responses from each of the two review authors and retrieved the full-text study reports/publications. Two review authors (of KR, NM, AT, LE, DW, AV, AD, LH) independently screened the full text and identified studies for inclusion and exclusion using the pre-specified inclusion criteria. In the case of any disagreements, a third author arbitrated (KR).

### Data abstraction

We used a data collection form for study characteristics and outcome data, which we had piloted. Two review authors (of KR, LE, DW, AV, AD, LH) extracted study characteristics and outcome data from included studies. Disagreements were resolved by consensus or by involving a third person (KR). One review author (KR) transferred data into the Review Manager software [31]. We double-checked that data were entered correctly by comparing the data presented in the systematic review with the data extraction form.

### Assessment of risk of bias in included studies

Two review authors (of KR, LE, DW, AV, AD, LH) independently assessed risk of bias for each study using the criteria outlined in the Cochrane Handbook for Systematic Reviews of Interventions [32]. We resolved any disagreements by discussion or by involving another author (KR). We assessed the risk of bias according to the following domains: random sequence generation, allocation concealment, blinding of participants and personnel, blinding of outcome assessment, incomplete outcome data, selective outcome reporting and other bias. We graded each potential source of bias as high, low or unclear.

### Measures of treatment effect

We processed data in accordance with the Cochrane Handbook for Systematic Reviews of Interventions [32]. We expressed dichotomous outcomes as risk ratios (RR) with 95% confidence intervals (CI). Where available we used adjusted estimates of treatment effect as hazard ratios, and used the inverse variance method to pool these statistically. For continuous variables, we compared net changes (i.e. intervention group minus control group differences) and calculated mean differences (MD) and 95% CIs for each study. For trials with multiple arms we divided the control group N by the number of arms to avoid double-counting in meta-analyses. We analysed outcomes at the longest period of follow-up where multiple measurements had been taken unless there was significant (> 30%) attrition. We narratively described skewed data reported as medians and interquartile ranges.

### Data synthesis

We summarised and analysed all eligible studies in Review Manager 5. We undertook meta-analyses only where this was meaningful, i.e. if the treatments, participants and the underlying clinical question were similar enough for pooling to make sense. We used a random-effects model as we could not assume that all studies in the meta-analysis are estimating the same intervention effect, but rather are estimating intervention effects that follow a distribution across studies. We used the I^2^ statistic to measure heterogeneity among the trials in each analysis. We used the five GRADE considerations (study limitations, consistency of effect, imprecision, indirectness and publication bias) to assess the quality of a body of evidence as it relates to the studies that contribute data to the meta-analyses for the prespecified outcomes. In the main analysis we did not combine primary and secondary prevention studies and different comparator groups as this would have made interpretation of the results difficult due to heterogeneity; instead we conducted four main analyses: Mediterranean dietary intervention versus no intervention or minimal intervention for primary prevention; Mediterranean dietary intervention versus another dietary intervention for primary prevention; Mediterranean dietary intervention versus usual care for secondary prevention; Mediterranean dietary intervention versus another dietary intervention for secondary prevention.

## Results

### Study selection and characteristics

**Figure 1:**
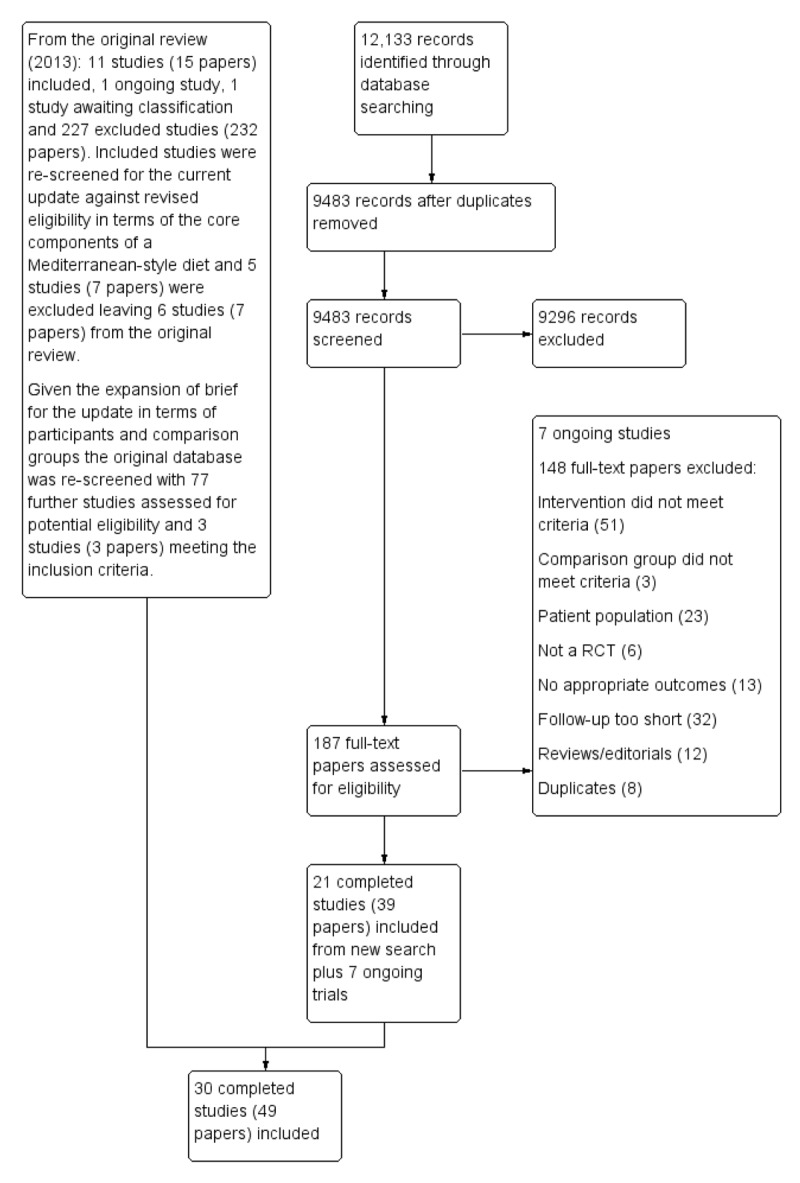
PRISMA flow for study selection.

Searching for this updated Cochrane review yielded 9483 records after duplicates were removed. Following screening of titles and abstracts 9296 were excluded and 187 full text papers were assessed for eligibility. Twenty one completed and seven ongoing trials were included from the new search. Six studies were included from the previous review and rescreening the original database identified three further trials, so a total of 30 completed studies and seven ongoing trials were included in this review (Figure 1).

The characteristics of the 30 completed included studies are shown in Table 1. Results are described for each of the four main analyses.

**Table 1:** Characteristics of included studies.

Study	Country	Sample Size	Participants	Mean age (years)	% male	Intervention	Comparison	Follow-up duration

**Athyros 2011 [33]**	?	150 (100 for this review)	Mild hyper-cholesterolaemia	54.7	49	Trained dieticians encouraged participants to adhere to a Mediterranean dietary pattern, with efforts to increase adherence and 7-day menu plans with food that incorporated the salient characteristics of the Mediterranean diet.	Hypolipidaemic diet	16 weeks
**Bajerska 2018 [34]**	Poland	144	Postmenopausal women with central obesity	60.5	0	Participants followed a food plan based on Mediterranean dietary recommendations. Typical Mediterranean food products were used providing approximately 37% energy from total fat, 20% from MUFAs, 9% from PUFAs, 8% from SFAs, 18% from protein and 45% energy from carbohydrates. Olive oil was used in every meal and 5 to 7 nuts were served once a day.	Central European diet	4 months
**Castagnetta 2002 [35]**	Italy	115	Healthy postmenopausal women	44 to 71	0	MEDIET project - participants were invited to a weekly cooking course and a social dinner with chefs addressing the principles of the traditional Mediterranean diet. The proposed recipes were based on a traditional Sicilian diet including whole cereals, legumes, seeds, fish, fruits, vegetables, olive oil and red wine to consume on a daily basis at home. Women were asked to avoid refined carbohydrates, salt and additional animal fat.	Usual diet	6 and 12 months
**Chasapidou 2014 [36]**	Greece	384	Know cardiometabolic diseases	?	?	Mediterranean healthy diet personalised in calories and nutrients according to the patient’s cardiometabolic disease, monthly follow-up by a dietitian.	No dietary counselling	6 months
**Clements 2017 [37]**	UK	120	Healthy elderly participants	70	39	Dietary advice sheets and individual dietary advice to achieve the quantitative requirements for the Nu-AGE dietary intervention. Participants were given extra-virgin olive oil, whole grain pasta and low-fat margarine rich in MUFA and PUFA freely throughout the study.	Standard healthy living advice leaflet from the British Dietetic Association and asked to maintain habitual dietary intake.	1 year
**Colquhoun 2000 [38]**	?	68	Documented CHD	?	?	Mediterranean diet: 35% to 40% energy from fat with > 50% of fat being monounsaturated	Low-fat diet: 20% to 25% energy from fat with 8% to 10% saturated	3 months
**Davis 2017 [39]**	Australia	166	Healthy elderly participants	71	44	Based on a traditional Mediterranean diet, with small adaptations to the Australian food supply. Resources were provided that included a recipe book, guidelines for eating out, serving sizes and the recommended number of servings, and participants also received foods (olive oil, nuts, legumes, tuna and Greek yogurt) to increase the likelihood of adherence.	Regular diet without change (seasonal variation permitted). Participants received a voucher to buy regularly consumed foods from supermarkets.	6 months
**Dinu 2017 [40]**	?	117	Overweight with at least an additional metabolic risk factor	51	15	Mediterranean diet of 3 different sizes (1400, 1600, 1800 Kcal/day), according to specific energy requirements.	Vegetarian diet of 3 different sizes (1400, 1600, 1800 Kcal/day), according to specific energy requirements.	3 months in each phase (cross-over)
**Djuric 2009 [41]**	US	69	Healthy non-obese women	44	0	Greek Mediterranean exchange list diet with exchange goals determined by dieticians at baseline and focused on increasing fruit and vegetable intake and variety and increasing MUFA intake while maintaining the baseline energy intake and total fat intake. Participants were given 3 L of extra-virgin olive oil at baseline and at 3 months.	Usual diet with no counselling, but participants were given the National Cancer Institutes Action guide to healthy eating and written materials on nutritional deficiencies if below 67% RDA.	6 months
**Entwistle 2018 [42]**	UK	41	Heart and lung transplant recipients	58	70	Information and encouragement to follow an eating pattern representative of a traditional Mediterranean diet. The key dietary recommendations were: daily mixed consumption of a range of vegetables, fruit, whole grains, fish/seafood, raw nuts and legumes; abundant use of extra-virgin olive oil (a free 5L container of extra-virgin olive oil was provided to each participant); moderate consumption of dairy products and red wine; low intake of red and processed meats, of sweets, sweet-baked pastries and sweetened beverages.	Modified British Heart Foundation low-fat guidelines, with an emphasis on consuming mainly plant-based whole foods similar to the Mediterranean diet, and advice to minimise high-fat foods. Each participant received a low-fat recipe book.	12 months
**Esposito 2004 [43]**	Italy	180	Metabolic syndrome	44	55	Advice about a Mediterranean-style diet. Through a series of monthly small-group sessions, participants received education in reducing dietary calories (if needed), personal goal-setting and self-monitoring using food diaries. Behavioural and psychological counselling was also offered. Dietary advice was tailored to each participant on the basis of 3-day food records. Participants were in the programme for 24 months and had monthly sessions with the nutritionist for the first year and twice monthly sessions for the second year.	Oral and written information about healthy food choices. The general recommendation for macro-nutrient composition of the diet was similar to that for the intervention group. Participants had bimonthly sessions with study personnel.	2 years
**Konstantinidou 2010 [44]**	Spain	90	Healthy adults	20 to 50	28	1. Traditional Mediterranean diet with virgin olive oil 2. Traditional Mediterranean diet with washed virgin olive oil The dietician gave personalised advice during a 30-minute session to each participant following the traditional Mediterranean diets, with recommendations on the desired frequency of intake of specific foods.	Control group (30 participants): participants were advised by a dietician to maintain their habitual lifestyle	3 months
**Lapetra 2018 [45]**	Spain	180	Hypertensive patients	55 to 75	92	Mediterranean-style diet Both groups received dietary advice (individual and group) every 3 months for at least 2 years. Participants attended educational talks about hypertension and healthy eating and were given a booklet that included essential information from the talks and a seasonal menu, tailored for each group.	Low-fat diet according to American Heart Association guidelines	2 years
**Lindman 2004 [46]**	Norway	219 in whole study, 98 for the arms of interest to this review	Longstanding hyper-cholesterolaemia	69.7	100	Dietary advice (‘Mediterranean-type’ diet) and placebo capsules. Diet counselling was given individually by a clinical nutritionist based on a food frequency questionnaire. Participants were supported with a margarine rich in PUFA and vegetable oils free of cost. 2 placebo capsules were taken twice daily corresponding to 2.4 g corn oil.	Usual care + placebo capsules. 2 placebo capsules were taken twice daily corresponding to 2.4 g corn oil.	6 months
**Mayr 2018 [47]**	Australia	73	Documented CHD patients	62	84	Based on a traditional Cretan Mediterranean diet. Modelled a 2-week meal plan incorporating key dietary components of a Mediterranean diet with a mix of traditional and modified recipes considered to be realistic options for multi-ethnic Australians. Patients received a recipe book, shopping lists, a food pyramid, weekly dietary intake checklists and label reading information. Hampers were provided at baseline and 3 months to aide adherence (6 L extra-virgin olive oil, 1.2 kg nuts, tinned fish and legumes and Greek yogurt).	Low-fat diet - followed the standard diet recommendations for cardiac patients in Australia at the time. A one week meal plan was provided, resources for label reading, low-fat cooking and recommended food group serving sizes. Participants received a supermarket voucher at the 3 face-to-face meetings.	6 months
**Michalsen 2006 [48]**	Germany	101	Documented CHD patients	59	77	A lifestyle modification group with the focus on Mediterranean diet: 3-day non-residential retreat, and regular group meetings thereafter. The lifestyle programme addressed diet and stress management. Participants were extensively informed about the Mediterranean diet by nutritional information, repetitive group discussions, cooking classes and group meals, and dietary instructions were tailored to individuals where necessary.	Patients in the control group received only written and less detailed information about the dietary principles of the Mediterranean diet, and some leaflets with general advice about stress reduction.	1 year
**Misciagna 2017 [49]**	Italy	98	Non-alcoholic fatty liver disease	?	50	Low glycaemic index Mediterranean diet (LGIMD).	Italian National Research Institute for Foods and Nutrition (INRAN) guidelines, with information provided in brochures using in a traffic-light format.	6 months
**Ng 2011 [50]**	China (Hong Kong)	48	HIV patients	?	?	The dietitian designed an individualised meal plan for each participant, taking into account any specific requirements related to their HIV status. The Mediterranean diet was modified to suit the local culture, for example: replacing red meat with fish or chicken; using canola, rapeseed or olive oil in place of cooking oil to replace saturated fats; canola margarine in place of butter or other margarine; using dried legumes or tofu to replace meat; using low-fat dairy or soy drink instead of full fat dairy.	Low-fat and low-cholesterol diet prescribed according to the NCEP Adult Treatment Panel III guidelines.	12 months
**PREDIMED [51]**	Spain	7447	Increased risk of CVD (either T2DM or 3 or more risk factors)	67	42	1) Mediterranean diet + extra-virgin olive oil 2) Mediterranean diet + tree nuts Advice to adhere to a Mediterranean diet through tailored individual visits to dieticians and group sessions every 3 months. Group sessions included informative talks and discussion with review of dietary goals, menu planning and shopping lists appropriate for each dietary intervention and provision of supplemental extra-virgin olive oil or nuts.	Low-fat diet – advice to reduce all types of fat, and recommending the consumption of lean meats, low-fat dairy products, cereals, potatoes, pasta, rice, fruits and vegetables. The use of olive oil was discouraged. Tailored individual visits to dieticians and group sessions every 3 months (after year 3 to match intensity of intervention). Non-food incentives provided at group sessions.	4.8 years
**Properzi 2018 [52]**	Australia	56	Non-alcoholic fatty liver disease	?	?	Mediterranean diet	Low-fat diet	12 weeks
**Singh 1992 [53]**	India	406	Definite or possible acute MI and unstable angina	51	90	Diet A - meat, eggs, hydrogenated oils, butter and clarified butter were replaced with vegetarian meat substitutes and soya bean, sunflower and ground nut oils. Patients were also advised to eat fruit, vegetables, pulses, nuts and fish. Patients following diet A had the advice regularly reinforced.	Diet B- meat, eggs, hydrogenated oils, butter and clarified butter were replaced with vegetarian meat substitutes and soya bean, sunflower and ground nut oils. Initial advice only, with no further reinforcement.	1 year (blood measures) and 2 years (clinical endpoints)
**Singh 2002 [54]**	India	1000	Risk factors for CVD	48.5	90	National Cholesterol Education Program (NCEP) step I prudent diet, plus Indo-Mediterranean diet - at least 400 to 500g of fruits, vegetables and nuts per day, 400g to 500g of whole grains, legumes, rice, maize and wheat daily, as well as mustard seed or soy bean oil, in 3 to 4 servings per day, which is consistent with recommendations from the Indian Consensus Group.	Control patients were given an information sheet on the National Cholesterol Education Program (NCEP) step I prudent diet.	2 years
**Skouroliakou 2017 [55]**	Greece	70	Women with breast cancer	?	0	Personalised dietary intervention based on the Mediterranean diet, conducted by 2 trained registered dietitians. The diet was enriched with olive oil and foods with specific health benefits for breast cancer survivors. They received a personalised dietary programme via e-mail as well as face-to-face appointments every 15 days for the first 3 months and phone calls at the end of months 4 and 5 with in-person meetings at the end of the study at 6 months. Specific meals, products, recipes and food portions, educational booklets, food diaries and individual nutritional advice was provided.	Received the updated American Cancer Society Guidelines on Nutrition and Physical Activity for Cancer Prevention and ad libitum diet. Patients were contacted by phone every 15 days for the first 3 months, then at months 4 and 5 and in-person meetings at baseline, 3 and 6 months.	6 months
**Sofi 2018 [56]**	Italy	118	Clinically healthy with low to moderate cardiovascular risk profile	Median age 50 (range 21 to 75)	22	Advice to follow a Mediterranean diet delivered through face-to-face individual counselling sessions. Participants were provided with a detailed 1-week menu plan as well as tips and information on the food groups that could be included and those that could not.	Advice to follow a lacto-ovo vegetarian diet delivered through face-to-face individual counselling sessions. Participants were provided with a detailed 1-week menu plan as well as tips and information on the food groups that could be included and those that could not. Included also recipes for preparing meals.	Cross-over at 3 months
**Stradling 2018 [57]**	UK	60	HIV patients on antiretroviral therapy with LDL >3mmol/L	?	?	Advice and support to adopt the Mediterranean diet, supplemented by additional functional foods with cholesterol-lowering properties. Motivational interviewing-style consultation.50 mL cholesterol-lowering drink at randomisation and subsequent sessions. Supplies of the functional foods (nuts, soy protein, plant stanols, oats and pulses) were given to participants to offset the additional cost of making dietary changes.	Wait-list control, with low saturated fat diet. Focus on reduction of saturated fat to < 10% of energy intake, in line with UK guidelines. Resources were provided, such as written information, recipes and online videos.	12 months
**The Lyon Diet Heart Study [58]**	?	605	MI within 6 months	53.5	90	Tailored advice during 1 hour session with research cardiologist and dietician to follow a Mediterranean-type diet. Further dietary counselling at each visit at 2 months and annually. A rapeseed (canola) oil-based margarine was supplied free for the whole family, as participants would not accept olive oil as the only fat.	No dietary advice apart from that of hospital dieticians or attending physicians as usual care.	24 and 46 months
**Tuttle 2008 [59]**	?	101	Recruited 6 within 6 weeks of first MI	58	74	Mediterranean-style diet, with emphasis on the increased consumption of cold-water fish (3 to 5 times/week) and oils from olives, canola and soybeans. Participants procured and prepared their own meals. Participants received individual dietary counselling sessions and group sessions focused on behavioural modification and practical aspects of their assigned diet, including recipes, grocery shopping and dining out.	Low-fat diet (the American Heart Association Step II diet). Participants received individual dietary counselling sessions and group sessions focused on behavioural modification and practical aspects of their assigned diet, including recipes, grocery shopping and dining out.	2 years
**Vincent-Baudry 2005 [60]**	France	212	At least one cardiovascular risk factor	51	41	Mediterranean diet - dietary advice given by physicians and dieticians, and participants received a booklet with nutritional recommendations. In addition, participants were provided with oat-bran enriched pasta, tomato sauce and olive oil.	Low-fat American Heart Association–type diet was adapted for the low-fat diet group.	3 months
**Wardle 2000 [61]**	UK	117	Mild to moderate hyper-cholesterolaemia	53.5	43.5	Advice to follow a Mediterranean diet delivered in 8 sessions during the 12-week intervention period using a combination of individual and group sessions with a dietician and psychologist. Advised to increase intake of fruit and vegetables, and oily fish and to reduce fat to 30% of energy with substitution of predominantly monounsaturated fat for saturated fat. Individualised advice to implement dietary changes based on their lifestyle and food preferences and group support in maintaining changes. Intervention participants were also given free spreading fats and oils high in monounsaturated fats.	Wait-list control with no specific dietary advice.	12 weeks
**Weber 2012 [62]**	Brazil	122	Established or previous CVD within past 10 years at increased CVD risk	63	66	Group B Received the dietary therapy that was proposed by the Brazilian guidelines for cardiovascular diseases, customised by the integration of typical Mediterranean foods (e.g. olives, olive oil, chestnuts, walnuts, almonds, hazelnuts, peanuts and cold water fish). Weekly counselling sessions with dietitians (in person or by telephone). Group C Received the same dietary intervention as Group B, but the patients were counselled monthly in person.	Group A Brazilian Cardioprotective Diet Program, i.e. avoiding high energy density foods (> 1 kcal/g); prioritisation of regional foods that are culturally accepted by the patients (rice, bean, soy oil, and Brazilian fruits and vegetables). Weekly in-person sessions with dietitians - tips for eating in restaurants, instructions on label reading and a list of typical Brazilian recipes that were adjusted for nutrients and energy densities.	12 weeks

Key: CHD (coronary heart disease), CVD (cardiovascular disease), HIV (human immunodeficiency virus), LDL (low-density lipoprotein), MI (myocardial infarction), MUFA (monounsaturated fatty acids), PUFA (polyunsaturated fatty acids), SFA (saturated fatty acids), T2DM (Type 2 Diabetes Mellitus).

#### 1. Mediterranean dietary intervention versus no intervention or minimal intervention for primary prevention

Nine trials were included with 1337 participants randomised. The majority of participants were classified as healthy and were recruited by three of the trials [35,41,44], with two further trials recruiting elderly people [37,39]. The remaining four trials recruited participants at increased cardiovascular risk [36,43,46,61]. Two trials recruited only women [35,41], and one only men [46]. The trials were conducted in the US [41], Italy [35,43], Spain [44], Greece [36], Norway [46], Australia [39], and the UK [37,61]. The duration of the intervention and follow-up periods varied: three months [44,61], six months [35,36,39,41,46], one year [37], and two years [43].

#### 2. Mediterranean dietary intervention versus another dietary intervention for primary prevention

Thirteen trials were included with 8687 participants randomised. The majority of participants were enrolled in one large multicentre trial with 7747 participants [51]. The majority of participants were described as at increased cardiovascular risk [33,34,40,42,45,49,50,51,52,56,57,60]. One study recruited women with breast cancer [55], two trials recruited only women [34,55]. The trials were conducted in Spain [45,51], Italy [40,49,56], Greece [33,55], France [60], the UK [42,57], Poland [34], Australia 52], and China [50]. The duration of the intervention and follow-up periods varied: three months [40,52,56,60], four months [33,34], six months [49,55], one year [42,50,57], two years [45], and up to five years [51]. The dietary interventions in the comparison group varied, including low-fat [33,42,45,50,51,52,57,60], the traditional diet of that country [34], national recommendations/disease-specific guidance [49,55], and vegetarian [40,56].

#### 3. Mediterranean dietary intervention versus usual care for secondary prevention

Two trials were included with 706 participants randomised. Both trials recruited patients with CVD, one in men and women with CHD [48], and the other in men and women who had experienced a myocardial infarction within six months [58]. Participants were recruited from Germany [48], and France [58]. The duration of the intervention and follow-up periods varied from 12 months to 24 and 46 months [48,58].

#### 4. Mediterranean dietary intervention versus another dietary intervention for secondary prevention

Six trials were included with 1731 participants randomised. An expression of concern has been published about the reliability of two of the studies in this comparison group with the majority of participants [53,54,63,64], and we have conducted sensitivity analyses excluding these studies from all analyses. All trials recruited patients with CVD. Three trials recruited men and women with CHD [38,47,62], one after a first myocardial infarction [59] and one with acute myocardial infarction or unstable angina [53]. One trial recruited patients with established CHD or those at high risk of CHD, although the majority of participants had established disease [54]. Participants were recruited from Australia [38,47], the US [59], Brazil [62], and India [53,54]. The duration of the intervention and follow-up periods varied: three months [38,62], six months [47], and two years [53,54,59]. The dietary interventions in the comparison group varied, including low-fat [38,47,59], and national recommendations/disease-specific guidance [53,54,62].

### Risk of bias in included studies

**Figure 2:**
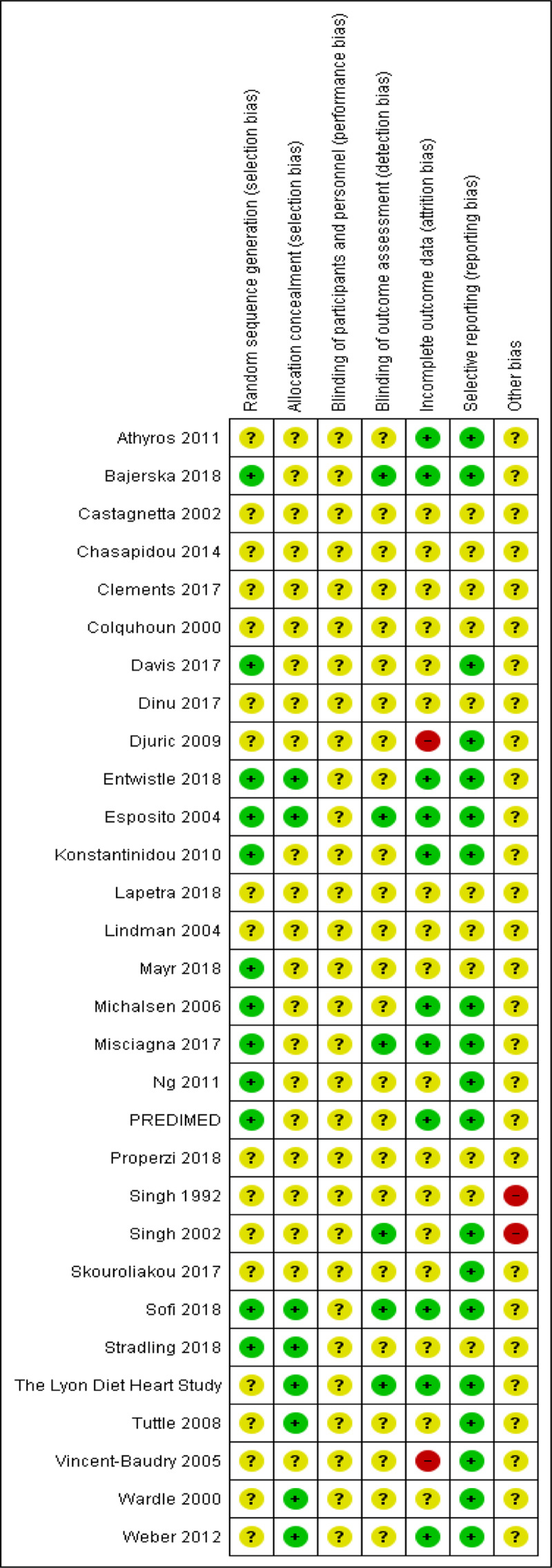
Risk of bias assessment of included studies.

The risk of bias of included studies is shown in Figure 2. The blinding of participants and personnel for behavioural interventions is difficult, if not impossible, in most cases and so we have not judged this as a high risk of bias. We rated this domain as unclear for all trials in all four comparison groups.

#### 1. Mediterranean dietary intervention versus no intervention or minimal intervention for primary prevention

The methods of random sequence generation were unclear in six of the nine included studies [35,36,37,41,46,61], and we judged the methods used to be at low risk of bias in the remaining three [39,43,44]. The methods of allocation concealment were unclear in seven studies and at low risk of bias in the remaining two [43,61]. Blinding of outcome assessment was unclear in eight trials, in the remaining trial we judged this to be at low risk of bias [43]. We judged three trials to be at low risk of attrition bias [43,44,61], one study was at high risk of bias as there was differential loss to follow-up [41], and the remaining trials were judged as unclear. The risk of bias associated with selective reporting was judged as low risk in five trials [39,41,43,44,61], and unclear in the remaining four. There was insufficient information to judge the risk of other sources of bias and we categorised all nine studies as unclear.

#### 2. Mediterranean dietary intervention versus another dietary intervention for primary prevention

The methods of random sequence generation were unclear in six of the 13 included studies [33,45,40,52,55,60], and judged at low risk of bias in the remaining seven [34,42,49,50,51,56,57]. The methods of allocation concealment were unclear in 10 studies and at low risk of bias in the remaining three [42,56,57]. Blinding of outcome assessment was unclear in 10 of the 13 trials and judged at low risk of bias in the remaining three [34,49,56]. We judged six of the 13 trials to be at low risk of attrition bias [33,34,42,49,51,56], one study to be at high risk of bias for attrition due to differential loss to follow-up [60], and unclear in the remaining six trials. The risk of bias associated with selective reporting was unclear in four studies and judged to be of low risk of bias for the remaining nine [33,34,42,49,50,51,55,56,60]. There was insufficient information to judge the risk of other sources of bias and we categorised all 13 studies as unclear.

#### 3. Mediterranean dietary intervention versus usual care for secondary prevention

The methods of random sequence generation were unclear in one of the two included studies [58], and in the other we judged the methods used to be at low risk of bias [48]. The methods of allocation concealment were unclear in one study [48], and at low risk of bias in the other [58]. Blinding of outcome assessment was unclear in one trial at low risk of bias for the other [48,58]. We judged both trials to be at low risk of attrition bias and selective reporting. There was insufficient information to judge the risk of other sources of bias and we categorised both studies as unclear.

#### 4. Mediterranean dietary intervention versus another dietary intervention for secondary prevention

The methods of random sequence generation were unclear in five of the six included studies [38,53,54,59,62], and in the one study where this was clear, we judged the methods used to be at low risk of bias [47]. The methods of allocation concealment were unclear in four studies and at low risk of bias in the remaining two [59,62]. Blinding of outcome assessment was unclear in five studies and judged to be at low risk of bias in one [54]. The risk of attrition bias was unclear in all six trials. The risk of bias associated with selective reporting was unclear in three studies and at low risk of bias in three [54,59,62]. An expression of concern has been published about the reliability of two of the studies in this comparison group [53,54,63,64]. We have conducted sensitivity analyses excluding these studies from all analyses. We regarded these two studies as at high risk of other bias. We judged the remaining four studies as at unclear risk of other sources of bias as there was insufficient information to make a judgement.

## Effects of interventions

### CVD clinical events

We assessed the overall quality of evidence using the five GRADE considerations (study limitations, consistency of effect, imprecision, indirectness and publication bias) presented in Table 2 for each of the four main comparisons.

**Table 2:** Summary of Findings Table for primary outcomes for each of the four main comparison groups.

Comparison	Primary outcomes	RCTs (n)	Participants (n)	Follow-up (years)	Anticipated absolute effects (95% CI)		Effect estimate (95% CI)	Certainty of the evidence (GRADE)	Interpretation

					Control (events per 1000)	Intervention (events per 1000)			

Mediterranean diet vs no or minimal intervention (primary prevention)	Cardiovascular mortality	Not reported							
	Total mortality	Not reported							
Mediterranean diet vs another dietary intervention (primary prevention)	Cardiovascular mortality	1	7447	4.8	12	10 (6 to 16)	HR 0.81(0.50 to 1.32)	●●○○ LOW	Little or no effect
	Total mortality	1	7447	4.8	47	47 (38 to 57)	HR 1.00(0.81 to 1.24)	●●○○ LOW	Little or no effect
	Myocardial infarction	1	7447	4.8	16	12 (9 to 17)	HR 0.79(0.57 to 1.10)	●●○○ LOW	Little or no effect
	Stroke	1	7447	4.8	24	14 (11 to 19)	HR 0.60(0.45 to 0.80)	●●●○ MODERATE	Reduction
Mediterranean diet vs usual care (secondary prevention)	Cardiovascular mortality	1	605	3.8	63	22 (9 to 51)	RR 0.35(0.15 to 0.82)	●●○○ LOW	Reduction
	Total mortality	1	605	4	79	35 (17 to 73)	RR 0.44(0.21 to 0.92)	●●○○ LOW	Reduction
Mediterranean diet vs another dietary intervention (secondary prevention)	Total cardiac endpoints	1	101	2	160	157 (64 to 386)	RR 0.98(0.40 to 2.41)	●○○○ VERY LOW	Uncertainty

#### 1. Mediterranean dietary intervention versus no intervention or minimal intervention for primary prevention

None of the nine included studies reported on clinical events.

#### 2. Mediterranean dietary intervention versus another dietary intervention for primary prevention

The PREDIMED trial was the only trial reporting clinical events for this comparison [51]. This trial was retracted and re-analysed following concerns regarding randomisation at two of 11 sites and the inclusion of non-randomised second household members. Low-quality evidence shows little or no effect of the intervention (advice to follow a Mediterranean diet plus supplemental extra-virgin olive oil or tree nuts) compared to a low-fat diet on CVD mortality (hazard ratio [HR] 0.81, 95% confidence interval [CI] 0.50 to 1.32) or total mortality (HR 1.0, 95% CI 0.81 to 1.24) over 4.8 years. There was, however, a reduction in the number of strokes with the intervention (HR 0.60, 95% CI 0.45 to 0.80), moderate-quality evidence).

#### 3. Mediterranean dietary intervention versus usual care for secondary prevention

The Lyon Diet Heart Study examined the effect of advice to follow a Mediterranean diet and supplemental canola margarine compared to usual care in 605 CHD patients over 46 months and there was low-quality evidence of a reduction in adjusted estimates for CVD mortality (HR 0.35, 95% CI 0.15 to 0.82) and total mortality (HR 0.44, 95% CI 0.21 to 0.92) with the intervention [58].

#### 4. Mediterranean dietary intervention versus another dietary intervention for secondary prevention

Three studies reported clinical endpoints for this comparison group [53,54,59], and two of these were excluded in sensitivity analyses from all main analyses due to published concerns regarding the reliability of the data [53,54,63,64]. Only one small trial (101 participants) provided unadjusted estimates for composite clinical endpoints for comparison four (very low-quality evidence of uncertain effect) [59].

### Cardiovascular risk factors

**Figure 3:**
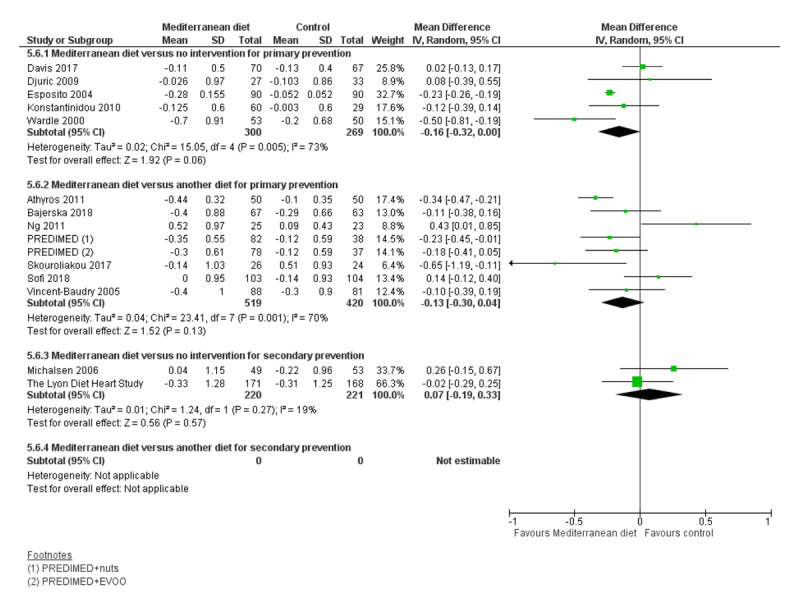
Total Cholesterol.

**Figure 4:**
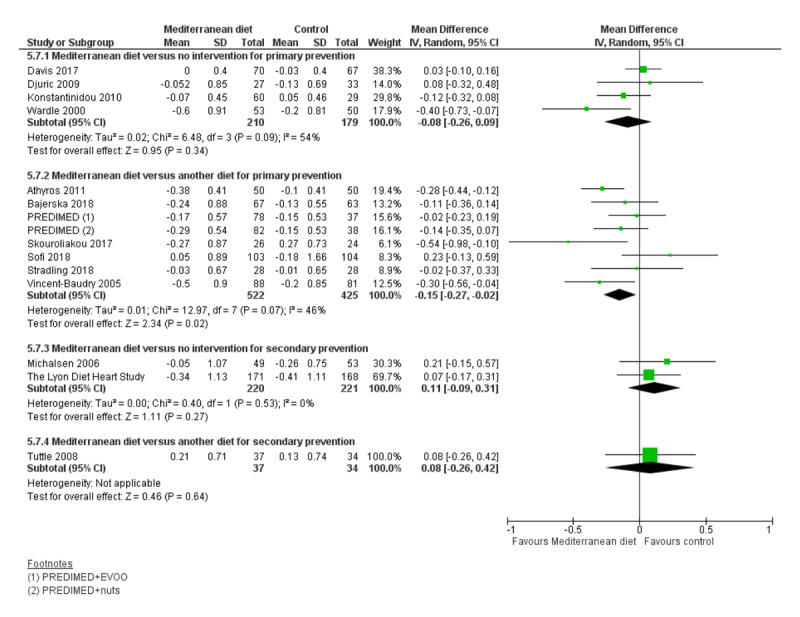
LDL Cholesterol.

**Figure 5:**
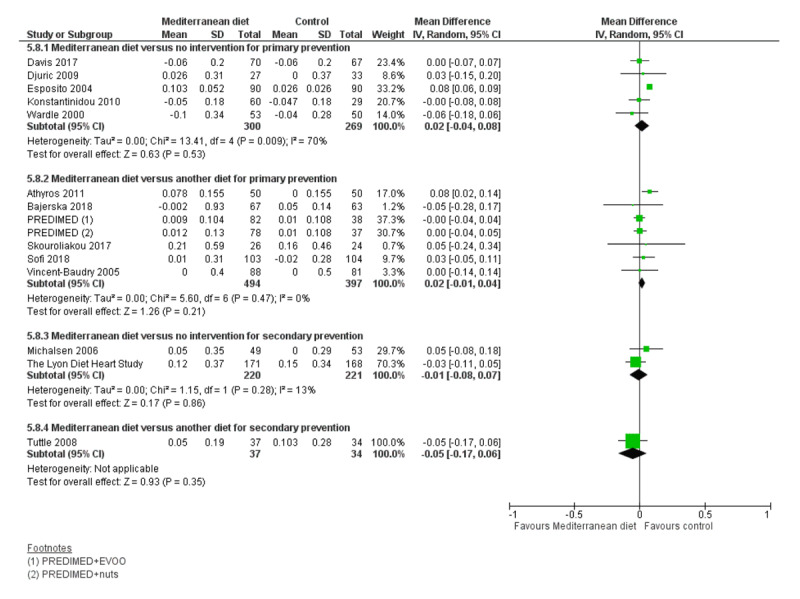
HDL Cholesterol.

**Figure 6:**
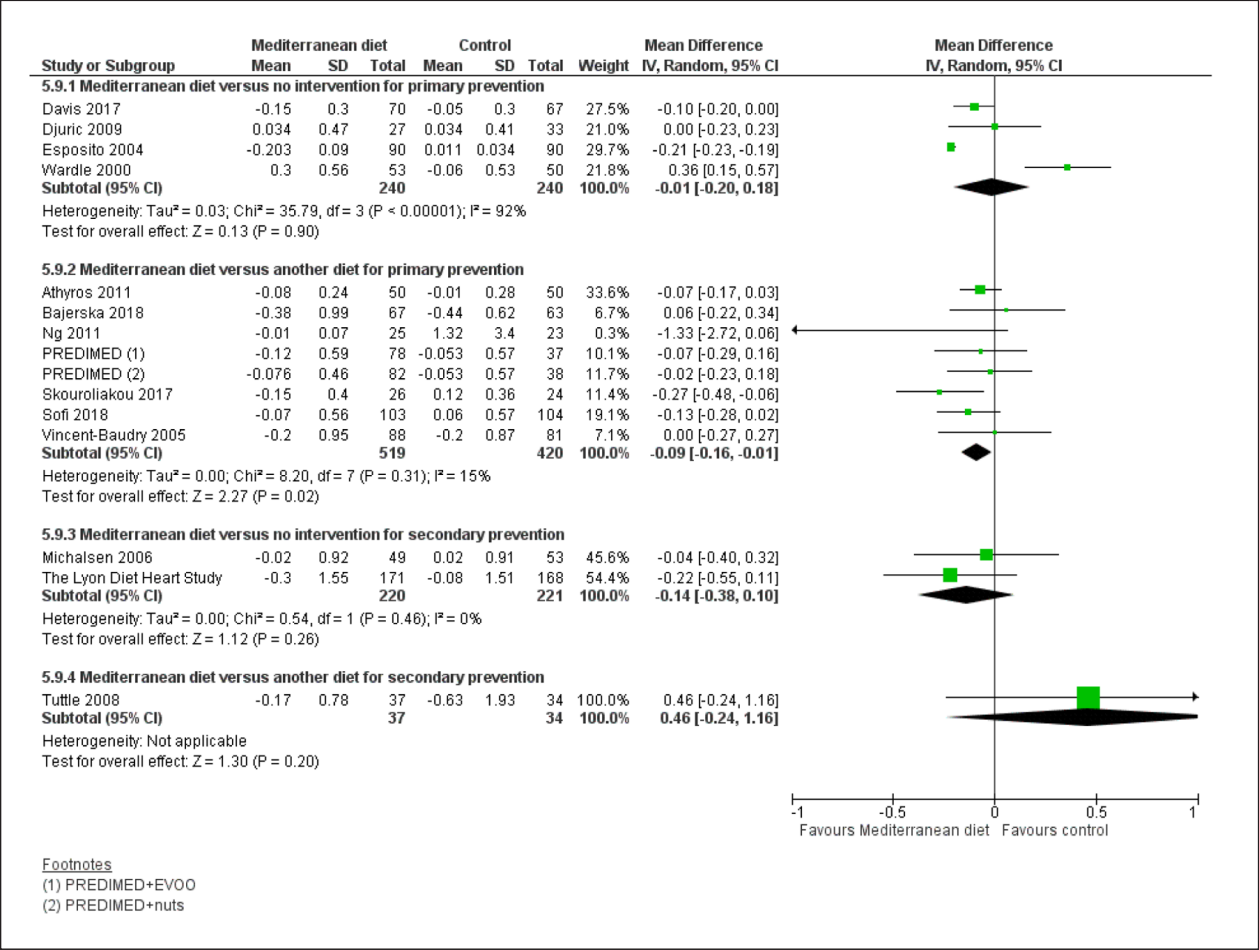
Triglycerides.

**Figure 7:**
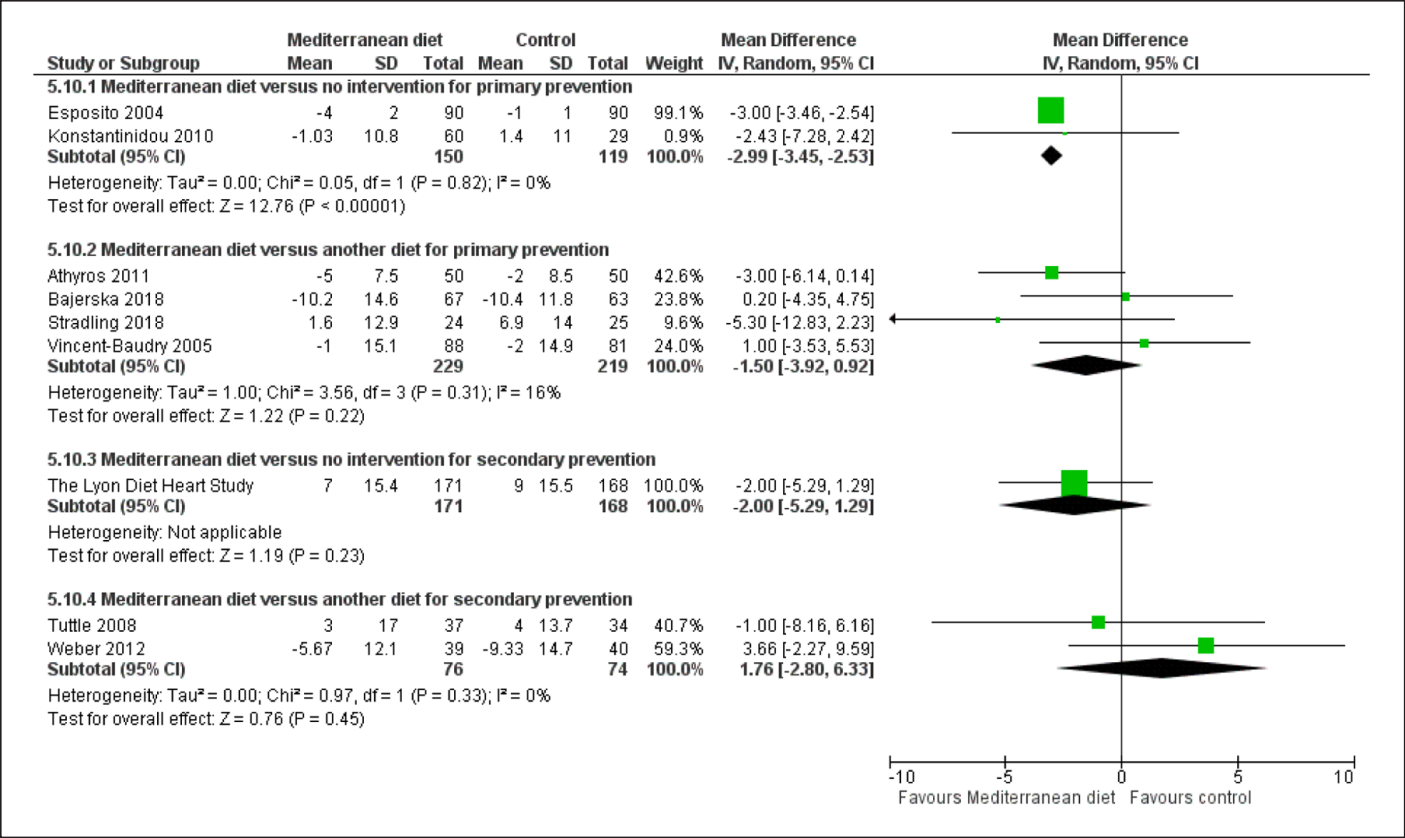
Systolic Blood Pressure.

**Figure 8:**
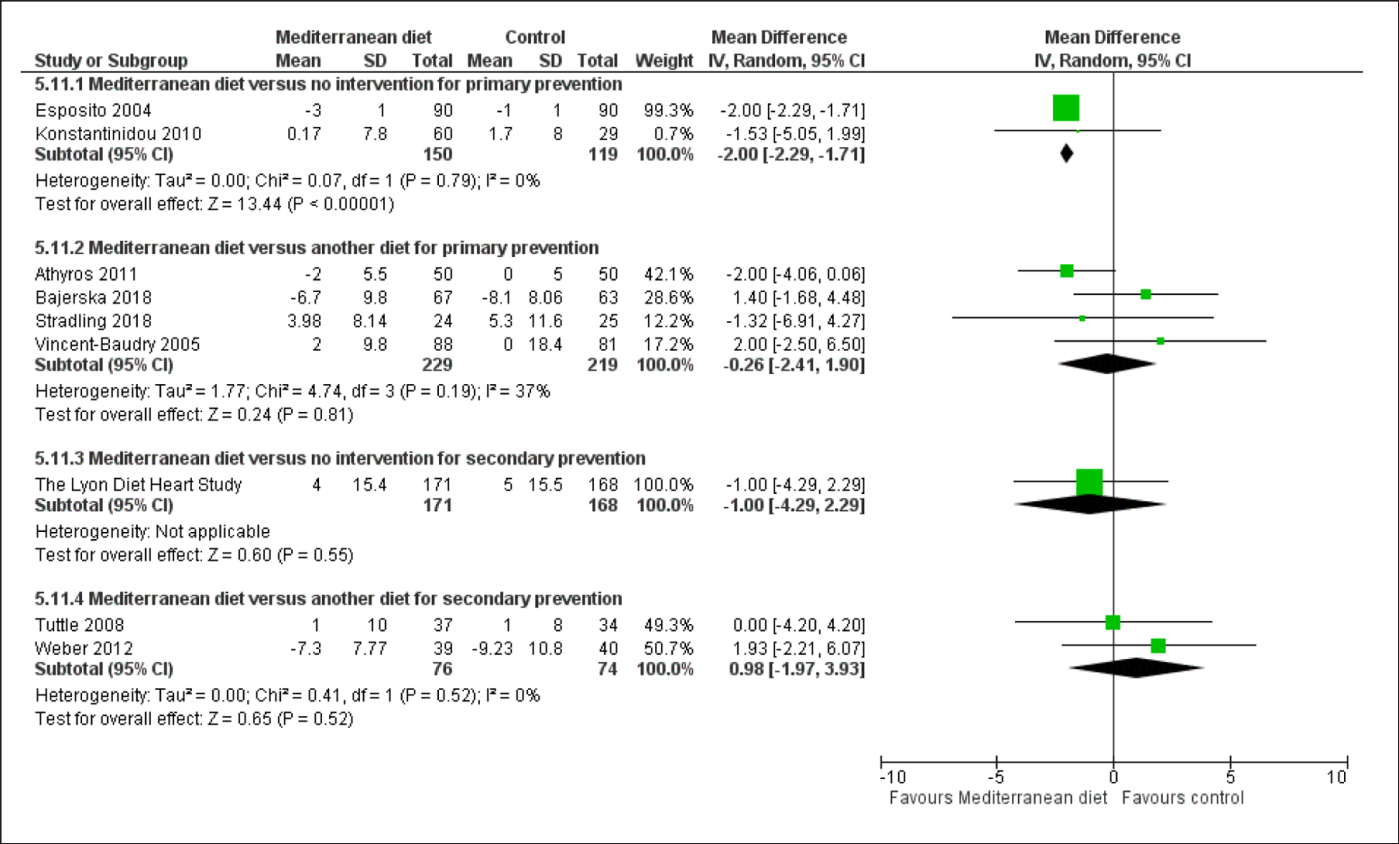
Diastolic Blood Pressure.

We assessed the overall quality of evidence using the five GRADE considerations (study limitations, consistency of effect, imprecision, indirectness and publication bias) presented in Table 3 for each of the four main comparisons. Figures 3, 4, 5, 6, 7, 8 show stacked forest plots for the four main comparisons for each of the following outcomes: Total cholesterol (Figure 3), LDL cholesterol (Figure 4), HDL Cholesterol (Figure 5), Triglycerides (Figure 6), Systolic Blood Pressure (Figure 7), Diastolic Blood Pressure (Figure 8).

**Table 3:** Summary of Findings Table for secondary outcomes for each of the four main comparison groups.

Comparison	Secondary outcomes, change from baseline	RCTs (n)	Participants (n)	Follow-up (months)	Mean change from baseline in control group (range)	Effect estimate (95% CI)	Certainty of the evidence (GRADE)	Interpretation

Mediterranean diet vs no or minimal intervention (primary prevention)	Total cholesterol (mmol/L)	5	569	3 to 24	–0.003 to –0.2	MD –0.16 (–0.32 to 0.00)	●●○○ LOW	Small reduction
	LDL cholesterol (mmol/L)	4	389	3 to 6	–0.2 to 0.05	MD –0.08 (–0.26 to 0.09)	●○○○ VERY LOW	Little or no effect
	HDL cholesterol (mmol/L)	5	659	3 to 24	–0.07 to 0.03	MD 0.02 (–0.04 to 0.08)	●●○○ LOW	Little or no effect
	Triglycerides (mmol/L)	4	480			Not pooled	●●○○ LOW	Little or no effect
	Systolic blood pressure (mmHg)	2	269	3 to 24	–1 to 1.4	MD –2.99 (–3.45 to –2.53)	●●●○ MODERATE	Reduction
	Diastolic blood pressure (mmHg)	2	269	3 to 24	–1 to 1.7	MD –2.00 (–2.29 to –1.71)	●●●○ MODERATE	Reduction
Mediterranean diet vs another dietary intervention (primary prevention)	Total cholesterol (mmol/L)	7	939	3 to 58	–0.29 to 0.51	MD –0.13 (–0.30 to 0.04)	●●○○ LOW	Little or no effect
	LDL cholesterol (mmol/L)	7	947	3 to 58	–0.18 to 0.27	MD –0.15 (–0.27 to –0.02)	●●●○ MODERATE	Small reduction
	HDL cholesterol (mmol/L)	6	891	3 to 58	–0.02 to 0.16	MD 0.02 (–0.01 to 0.04)	●●●○ MODERATE	Little or no effect
	Triglycerides (mmol/L)	7	939	3 to 58	–0.44 to 1.32	MB –0.09 (–0.16 to –0.01)	●●●○ MODERATE	Small reduction
	Systolic blood pressure (mmHg)	4	448	3 to 12	–10.4 to 6.9	MD – 1.50 (–3.92 to 0.92)	●●○○ LOW	Little or no effect
	Diastolic blood pressure (mmHg)	4	448	3 to 12	–8.1 to 5.3	MD –0.26 (–2.41 to 1.90)	●●○○ LOW	Little or no effect
Mediterranean diet vs usual care (secondary prevention)	Total cholesterol (mmol/L)	2	441	12 to 48	–0.22 to –0.31	MD 0.07 (–0.19 to 0.33)	●●○○ LOW	Uncertainty
	LDL cholesterol (mmol/L)	2	441	12 to 48	–0.26 to –0.41	MD 0.11 (–0.09 to 0.31)	●●○○ LOW	Uncertainty
	HDL cholesterol (mmol/L)	2	441	12 to 48	0 to 0.15	MD –0.01 (–0.08 to 0.07)	●●○○ LOW	Uncertainty
	Triglycerides (mmol/L)	2	441	12 to 48	–0.02 to –0.08	MD –0.14 (–0.38 to 0.10)	●●○○ LOW	Uncertainty
	Systolic blood pressure (mmHg)	1	339	48	9	MD –2.00 (–5.29 to 1.29)	●○○○ VERY LOW	Uncertainty
	Diastolic blood pressure (mmHg)	1	339	48	5	MD –1.00 (–4.29 to 2.29)	●○○○ VERY LOW	Uncertainty
Mediterranean diet vs another dietary intervention (secondary prevention)	Total cholesterol (mmol/L)*	Not reported						
	LDL cholesterol (mmol/L)*	1	71	24	0.13	MD 0.08 (–0.26 to 0.42)	●○○○ VERY LOW	Little or no effect
	HDL cholesterol (mmol/L)*	1	71	24	0.10	MD –0.05 (–0.17 to 0.06)	●●○○ LOW	Little or no effect
	Triglycerides (mmol/L)*	1	71	24	–0.63	MD 0.46 (–0.24 to 1.16)	●○○○ VERY LOW	Little or no effect
	Systolic blood pressure (mmHg)*	2	150	3 to 24	4 to –9.33	MD 1.76 (–2.80 to 6.33)	●○○○ VERY LOW	Little or no effect
	Diastolic blood pressure (mmHg)*	2	150	3 to 24	1 to –9.23	MD 0.98 (–1.97 to 3.93)	●○○○ VERY LOW	Little or no effect

*sensitivity analysis without Singh studies.

#### 1. Mediterranean dietary intervention versus no intervention or minimal intervention for primary prevention

For CVD risk factors for comparison one, there was low-quality evidence for a possible small reduction in total cholesterol (–0.16 mmol/L, 95% CI –0.32 to 0.00) and moderate-quality evidence for a reduction in systolic (–2.99 mmHg (95% CI –3.45 to –2.53) and diastolic blood pressure (–2.0 mmHg, 95% CI –2.29 to –1.71), with low or very low-quality evidence of little or no effect on LDL or HDL cholesterol or triglycerides.

#### 2. Mediterranean dietary intervention versus another dietary intervention for primary prevention

For comparison 2 there was moderate-quality evidence of a possible small reduction in LDL cholesterol (–0.15 mmol/L, 95% CI –0.27 to –0.02) and triglycerides (–0.09 mmol/L, 95% CI –0.16 to -0.01) with moderate or low-quality evidence of little or no effect on total or HDL cholesterol or blood pressure.

#### 3. Mediterranean dietary intervention versus usual care for secondary prevention

For comparison three there was low-quality evidence of little or no effect of a Mediterranean-style diet on lipid levels and very low-quality evidence for blood pressure.

#### 4. Mediterranean dietary intervention versus another dietary intervention for secondary prevention

For comparison four where only two trials contributed to the analyses there was low or very low-quality evidence of little or no effect of the intervention on lipid levels or blood pressure.

## Discussion

### Summary of main results

In this substantive review update, 30 RCTs (49 papers) and seven ongoing trials met our inclusion criteria. Four pre-specified comparison groups were used to analyse the data to address both heterogeneity between participants and comparison groups and aid interpretation of findings. Clinical endpoints were measured in only one large primary prevention trial (PREDIMED) [51], where following retraction and reanalysis of the data due to methodological concerns, there was low quality evidence of little or no effect of the intervention on total or CVD mortality or myocardial infarction but moderate quality evidence of a reduction in the number of strokes. For secondary prevention, in the Lyon Diet Heart Study [58], there was low quality evidence of reductions in total and CVD mortality and myocardial infarction. Two secondary prevention trials reporting clinical events were excluded from the main analyses due to published concerns regarding the reliability of the data [53,54,63,64]. A further small trial reported unadjusted estimates for total cardiac endpoints, with very low-quality evidence showing considerable uncertainly of the effect size. Cardiovascular risk factors including lipid levels and blood pressure were reported in all four comparison groups, with most of the studies contributing to primary prevention. Compared to no intervention or a minimal intervention, there was low-quality evidence for a possible small reduction in total cholesterol and moderate-quality evidence for a reduction in both systolic and diastolic blood pressure with a Mediterranean-style diet, with low or very low-quality evidence of little or no effect of the intervention on LDL or HDL cholesterol or triglycerides. Compared with other diets in primary prevention, there was moderate-quality evidence of a possible small reduction in LDL cholesterol and triglycerides with moderate or low-quality evidence of little or no effect of the intervention on total or HDL cholesterol or blood pressure. In secondary prevention few trials contributed to the analyses with low or very low-quality evidence of little or no effect of a Mediterranean-style diet on lipid levels or blood pressure.

### Study limitations and strengths

There were a large number of included trials (30 trials, 12,461 participants randomised), but few reported on clinical endpoints, our primary outcome, and the majority of trials reported on CVD risk factors for primary prevention. Due to the breadth of the review question, heterogeneity in terms of participants, interventions and comparators was high and we have attempted to reduce this by conducting the main analyses in four comparison groups for primary and secondary prevention and different comparators. Two studies were excluded from all main analyses in sensitivity analyses due to published concerns regarding the reliability of the data [53,54,63,64]. Only one trial reported clinical endpoints for primary prevention and this study experienced methodological issues regarding randomisation with the report subsequently being retracted and re-analysed [51]. The findings in secondary prevention are based on one older trial reporting very large effect estimates using a modified Zelen design [58]. Overall most trials were at unclear risk of bias for most domains and so results should be treated with caution.

We conducted a comprehensive search across major databases for interventions involving the Mediterranean diet. Two review authors independently selected and assessed trials for inclusion using pre-specified criteria, extracted data and assessed the quality of trials to minimise potential biases in the review processes. Our decision to restrict this review to interventions that only focused on the effectiveness of a Mediterranean-style diet per se avoided the potential confounding effects of other behavioural interventions on our outcomes, for example, those involving increased exercise in the context of trials examining multifactorial interventions. Our decision to exclude trials in people with diabetes who are at increased risk of CVD also missed relevant studies, but people with diabetes represent a distinct group and interventions for the management of diabetes are covered by another review group, the Cochrane Metabolic and Endocrine Disorders Group.

Definitions of the Mediterranean diet differed across studies. We used a classification system in our definition in an attempt to address the heterogeneity. The components required to meet our definition of a Mediterranean-style diet were based on previous definitions [13,14,15,16,17], and required at least the following two core components: high monounsaturated/saturated fat ratio (use of olive oil as main cooking ingredient and/or consumption of other traditional foods high in monounsaturated fats such as tree nuts) and high intake of plant-based foods, including fruits, vegetables and legumes. The rationale for this definition is based on recent work [5,30], which emphasises that the protective effects of the diet appear to be most attributable to olive oil, fruits, vegetables and legumes.

### Comparison with similar studies

Several recent systematic reviews and overviews of reviews have reported on the effects of the Mediterranean diet on cardiovascular health. A recent narrative overview of both prospective observational studies and RCTs concluded that the Mediterranean diet has some beneficial effects for CVD prevention but the effects were inconsistent between studies with few studies reported in meta-analyses and the overview authors called for more high-quality trials to address the inconsistencies [65]. This is in line with the findings of the current review reporting on RCT evidence. An umbrella review of systematic reviews reported on 13 meta-analyses of observational studies and 16 meta-analyses of RCTs investigating the association between the adherence to the Mediterranean diet and a number of different health outcomes [4]. The authors found robust evidence for a greater adherence to the Mediterranean diet and a reduced risk of overall mortality, cardiovascular diseases, coronary heart disease, myocardial infarction and diabetes with no evidence for LDL cholesterol levels.

A recent systematic review included both primary and secondary prevention trials and pooled clinical endpoints for these showing beneficial effects for major vascular events (risk ratio (RR) 0.69, 95% CI 0.55 to 0.86) and stroke (RR 0.66, 95% CI 0.48 to 0.92) [66]. A systematic review comparing the effects of a Mediterranean diet with low-fat diets on CVD risk factors in those at high risk or with established disease found favourable but modest effects of the Mediterranean diet on a wide range of cardiovascular risk factors and inflammatory markers, such as body weight, systolic and diastolic blood pressure, fasting plasma glucose, total cholesterol and high-sensitivity C-reactive protein [67]. Other systematic reviews have pooled together the evidence from both observational studies and RCTs on the effects of the Mediterranean dietary pattern on metabolic syndrome and individual cardiovascular risk factors, supporting favourable effects of the Mediterranean diet on cardio-metabolic risk factors [10,11]. The results of the current review including only RCTs show inconsistencies between studies but where meta-analyses were possible there were small beneficial effects on some CVD risk factors for primary prevention.

## Conclusions

Despite the large number of trials included in the review there is still uncertainty regarding the effects of a Mediterranean-style diet on clinical endpoints and cardiovascular disease (CVD) risk factors for both primary and secondary prevention from current clinical trial evidence. Two trials reporting clinical endpoints for secondary prevention were excluded because of concerns regarding the reliability of the data, so the available evidence is restricted to one large trial and a small trial reporting unadjusted estimates of effect. Evidence for primary prevention on clinical endpoints is limited to one large trial with methodological issues (although these have now been addressed in a recent re-analysis). Further adequately powered primary prevention trials are needed to confirm findings on clinical endpoints to date. Many trials reported on CVD risk factors, particularly in primary prevention, but heterogeneity precluded meta-analyses for some outcomes. Several ongoing trials have been identified which will add to the evidence base.

## Additional File

The additional file for this article can be found as follows:

10.5334/gh.853.s1 Appendix 1. Medline search strategy (Ovid).
