# Supplementary material for: Mediterranean-Style Diet for the Primary and Secondary Prevention of Cardiovascular Disease: A Cochrane Review
Source: Glob Heart. 2020 Aug 12;15(1):56. doi: 10.5334/gh.853 (PMC7427685; doi:10.5334/gh.853)
Supplement: Appendix 1. — Medline search strategy (Ovid). [file gh-15-1-853-s1.pdf]

Appendix 1 - Medline search strategy (Ovid)

1. exp Fruit/
2. fruit*.tw.
3. exp Vegetables/
4. Vegetable Proteins/
5. vegetable*.tw.
6. exp Fabaceae/
7. fabaceae.tw.
8. bean*.tw.
9. legume*.tw.
10. Lycopersicon esculentum/
11. lycopersicon esculent*.tw.
12. tomato*.tw.
13. solanum lycopersicum.tw.
14. Nuts/
15. (nut or nuts).tw.
16. Bread/
17. bread*.tw.
18. exp Cereals/
19. cereal*.tw.
20. grain*.tw.
21. Solanum tuberosum/
22. solanum tuberosum.tw.
23. potato*.tw.
24. Seeds/
25. (seed or seeds).tw.
26. olive oil.tw.
27. Fatty Acids, Monounsaturated/
28. monounsaturated fat*.tw.
29. mono-unsaturated fat*.tw.
30. exp Seafood/
31. exp Fish Oils/
32. fish.tw.
33. seafood*.tw.
34. shellfish.tw.
35. or/1-34
36. ((high or more or increase* or elevat* or much or rais*) adj6 (intake or consumption or consume or eat* or amount*)).tw.
37. 35 and 36
38. exp Dairy Products/
39. exp Milk Proteins/
40. milk*.tw.
41. marg?rine*.tw.
42. butter*.tw.
43. dairy.tw.
44. cheese*.tw.
45. red meat*.tw.
46. processed meat*.tw.
47. yog?urt*.tw.

48. red wine*.tw.
49. or/38-48
50. ((low or little or medium or moderate or less or decrease* or reduc* or restrict*) adj6 (intake or consumption or consume or eat* or amount*)).tw.
51. 49 and 50
52. Diet, Mediterranean/
53. (mediterranean adj3 diet*).tw.
54. (mediterranean adj6 food*).tw.
55. (mediterranean adj6 nutrition*).tw.
56. (mediterranean adj6 eat*).tw.
57. ((diet* or food* or nutrit* or eat*) adj2 (pattern* or habit*)).tw.
58. Food Habits/
59. or/52-58
60. 37 or 51 or 59
61. exp Cardiovascular Diseases/
62. cardio*.tw.
63. cardia*.tw.
64. heart*.tw.
65. coronary*.tw.
66. angina*.tw.
67. ventric*.tw.
68. myocard*.tw.
69. pericard*.tw.
70. isch?em*.tw.
71. exp Stroke/
72. (stroke or stokes).tw.
73. cerebrovasc*.tw.
74. apoplexy.tw.
75. (brain adj2 accident*).tw.
76. ((brain* or cerebral or lacunar) adj2 infarct*).tw.
77. exp Hypertension/
78. hypertensi*.tw.
79. peripheral arter* disease*.tw.
80. ((high or increased or elevated) adj2 blood pressure).tw.
81. exp Hyperlipidemias/
82. hyperlipid*.tw.
83. hyperlip?emia*.tw.
84. hypercholesterol*.tw.
85. hypercholester?emia*.tw.
86. hyperlipoprotein?emia*.tw.
87. hypertriglycerid?emia*.tw.
88. isch?emi*.tw.
89. emboli*.tw.
90. arrhythmi*.tw.
91. thrombo*.tw.
92. atrial fibrillat*.tw.
93. tachycardi*.tw.
94. endocardi*.tw.
95. (sick adj sinus).tw.
96. exp Diabetes Mellitus/

97. diabet*.tw.
98. exp Hyperglycemia/
99. hyperglycemi*.tw.
100. (glucose adj2 intoleran*).tw.
101. exp Insulin Resistance/
102. (metabolic adj3 syndrome adj3 x).tw.
103. metabolic cardiovascular syndrome.tw.
104. dysmetabolic syndrome x.tw.
105. insulin resistan*.tw.
106. exp Arteriosclerosis/
107. exp Cholesterol/
108. cholesterol.tw.
109. "coronary risk factor*".tw.
110. Blood Pressure/
111. blood pressure.tw.
112. or/61-111
113. 60 and 112
114. randomized controlled trial.pt.
115. controlled clinical trial.pt.
116. randomized.ab.
117. placebo.ab.
118. clinical trials as topic.sh.
119. randomly.ab.
120. trial.ti.
121. 114 or 115 or 116 or 117 or 118 or 119 or 120
122. exp animals/ not humans.sh.
123. 121 not 122
124. 113 and 123
